# An Unusual Presentation of Herpes Simplex Virus Encephalitis

**DOI:** 10.1155/2012/241710

**Published:** 2012-09-06

**Authors:** Ray Boyapati, George Papadopoulos, James Olver, Michael Geluk, Paul D. R. Johnson

**Affiliations:** ^1^Department of Medicine, Austin Health, Studley Road, Heidelberg, VIC 3084, Australia; ^2^Department of Psychiatry, Austin Health, Studley Road, Heidelberg, VIC 3084, Australia; ^3^Department of Emergency Medicine, Austin Health, Studley Road, Heidelberg, VIC 3084, Australia; ^4^Departments of Infectious Diseases and Microbiology, Austin Health, Studley Road, Heidelberg, VIC 3084, Australia; ^5^Department of Medicine, The University of Melbourne, Melbourne, VIC 3010, Australia

## Abstract

We present a case of a 65-year-old man with an acute alteration in mental state that was initially diagnosed as a functional psychiatric condition. After extensive workup, herpes simplex virus type 1 (HSV-1) was detected in the patient's cerebrospinal fluid (CSF) by polymerase chain reaction (PCR), and he responded rapidly to treatment with acyclovir. The case illustrates the importance of actively excluding organic causes in such patients, the need to have a low threshold of suspicion for HSV encephalitis, and the central role of CSF PCR testing for the diagnosis of HSV encephalitis, even in the absence of CSF biochemical abnormalities.

## 1. Introduction

Herpes simplex virus (HSV) encephalitis has a high mortality rate without treatment [1] with a variable clinical presentation making fast and accurate diagnosis difficult but of critical importance. The classic presentation is of fever plus neurological symptoms [2] with CSF biochemical abnormalities and positive CSF PCR for HSV. Historically, brain biopsy was required for the diagnosis of HSV encephalitis but CSF PCR is now considered the gold standard [3, 4].

We describe an atypical case of HSV encephalitis (CSF PCR positive) presenting with acute psychosis and without the classic CSF biochemical findings. Organic delirium presenting with acute psychosis is not uncommon, and clinicians should be aware of the features that suggest an organic cause such as acute onset and lack of past history of psychiatric illness [5]. The patient recovered after being treated successfully with acyclovir.

## 2. Case Report

A 65-year-old man of eastern European background was transferred to our tertiary teaching hospital with decreased conscious state and behavioural changes. His past history included depression (without prior psychotic episodes), alcohol abuse, and hypertension. He had no family history of psychiatric illness. His only regular medications were Citalopram and Olmesartan.

Six weeks prior to presentation he had been incarcerated for breaching a restraining order. Two weeks after being incarcerated, he was noted to have a change in his mental state where he became aggressive, desecrated his cell with faeces, and developed urinary incontinence. He was transferred to an emergency department at another hospital where a diagnosis of a functional psychiatric disorder was made and the patient was returned to prison without investigation. Over the subsequent four weeks there was further deterioration in his mental state, and he was transferred to a forensic psychiatric facility.

At this facility, the patient became increasingly aggressive and disinhibited, requiring sedation and restraint. Following the oral administration of olanzapine (10 mg) and clonazepam (2 mg), the patient became drowsy and was transferred to our hospital for a second opinion.

On arrival, we noted a Glasgow Coma Score of 7, but other examination and vital sign observations were unremarkable, in particular, he was afebrile. Biochemistry and ChestX-raywere normal, and computed tomography (CT) scan of the brain with contrast showed multiple old small basal ganglia infarcts but no acute abnormalities. Magnetic resonance imaging (MRI) of the brain performed subsequently was distorted by motion artefact but was reported to be normal within these limitations.

Mental state examination revealed fluctuations in level of cooperation from quiet and smiling to marked irritability. The thought form was tangential, and the patient alternated languages between Serbian and English. Delusions of misidentification of staff were present, as were visual hallucinations. While orientation to time and short-term memory were intact, there was disorientation to place and marked attentional disturbance reflected by poor completion of the serial 7s test. A lumbar puncture was performed which demonstrated a protein of 0.4 g/L (RR, <0.45 g/L), glucose of 3.9 mmol/L (RR, 2.2–5.5 mmol/L), erythrocytes of 11, no leucocytes, and a negative gram stain. C Reactive Protein (CRP) rose from normal to 54 mg/L (RR, <20 mg/L). An acute delirium of unknown cause rather than psychosis was diagnosed on the basis of prominent fluctuations in attention, and the patient was commenced on empirical IV acyclovir.

Further serological testing including for human immunodeficiency virus, hepatitis B virus, and hepatitis C virus were negative. Polymerase chain reaction (PCR) on cerebrospinal fluid (CSF) for enterovirus, cytomegalovirus, varicella zoster virus, and herpes simplex virus type 2 (HSV-2) was negative. Cryptococcal antigen was not detected. HSV-1 PCR, performed on our behalf at our state infectious diseases reference laboratory, was positive. After commencing treatment, the patient showed significant improvement in behaviour and cognition, and after eight days, he was transferred to another hospital. CRP also dropped from a peak of 54 mg/L to normal. In total, he received twelve days of IV acyclovir and two days of oral acyclovir and was transferred back to prison on completion of treatment.

## 3. Discussion

Psychosis is not uncommon in organic delirium, and assuming patients with psychotic features have a functional psychiatric condition is a common pitfall. Clues to an organic cause include a rapidly fluctuating course, the absence of past or family history of psychotic illness, the presence of nonauditory hallucinations, and the lack of mood-congruent delusions [5]. Clinicians should always consider the possibility of treatable medical conditions when patients present with a new psychotic episode failure; to do so may lead to preventable adverse patient outcomes. 

Herpes simplex virus (HSV) can cause severe necrotising encephalitis with a high mortality rate (approximately 70%) without treatment [1] and is the most common cause of encephalitis requiring hospitalisation in Australia [6]. Almost all cases outside the neonatal period are caused by HSV-1 [7]. Although seropositivity for HSV-1 in the developed world approaches 70% [8], HSV encephalitis is a sporadic and rare disease thought to occur after reactivation and CNS invasion via the trigeminal nerve or the olfactory tract [9]. 

Typically patients with HSV encephalitis will have fever plus at least one of a series of neurological deficits [2] (Table 1). However, the clinical presentation is variable, often presenting the clinician with a dilemma: on the one hand the prior probability of HSV encephalitis may be low, but on the other hand, acyclovir treatment is both lifesaving and needs to be commenced early to ensure an optimal outcome. Diagnosis of herpes encephalitis once required brain biopsy or the detection during convalescence of herpes-specific intrathecal antibody [10]. The advent of PCR not only allows rapid and immediate confirmation but has progressively revealed a much wider spectrum of herpes-related syndromes than previously suspected [11]. Other possible clinical features suggestive of HSV encephalitis include headache, urinary and faecal incontinence, and behavioural syndromes including loss of emotional control, hypomania, Kluver-Bucy syndrome, and psychosis. There are many case reports in the literature of patients presenting with purely behavioural abnormalities (with or without psychosis) who are eventually diagnosed with HSV encephalitis and treated successfully with antiviral therapy [12, 13]. 

The diagnosis of HSV encephalitis relies primarily on CSF analysis. CSF biochemistry usually reveals a pleocytosis, high protein and erythrocyte levels with normal glucose levels. Although uncommon, normal CSF biochemistry has been reported in some cases of HSV encephalitis [14]. One study reported that 3% of brain biopsy confirmed cases of HSV encephalitis had completely normal CSF findings [2]. 

CSF PCR is now considered the gold standard for the diagnosis of HSV encephalitis [3, 4]. However, some laboratories reject requests for herpes PCR testing on CSF specimens which lack predetermined criteria including elevated cell counts [15], highlighting the important role that clinicians have in pursuing unusual but treatable conditions in individual patients. Prior to the PCR era, detection of intrathecal synthesis of HSV-specific IgG antibodies more than 10 days after onset of disease was one of the principal assays for HSV encephalitis diagnosis [16]. These antibodies often persist in the CSF for several months or years, and this assessment has been recommended in combination with CSF PCR testing, mainly to help improve sensitivity of detection [17]. HSV intrathecal antibody detection would have helped confirm the diagnosis in our patient, but, unfortunately, the patient refused a further lumbar puncture.

Despite the normal CSF and imaging results, our patient's clinical features, response to treatment as evidenced by improvement in behaviour, cognition and CRP, as well as the CSF PCR result, are convincing evidence of atypical HSV-1 encephalitis masquerading as an episode of acute psychosis.

This case report illustrates that HSV encephalitis can present in unusual ways and reinforces the need for a low index of suspicion and early empirical use of acyclovir until results of definitive laboratory tests are available.

## Figures and Tables

**Table 1:** Clinical Presentation of HSV Encephalitis.

90% will have fever plus at least one of:
(1) Altered conscious state
(2) Personality change
(3) Focal cranial nerve deficits
(4) Hemiparesis
(5) Dysphasia
(6) Ataxia
(7) Focal seizures
